# A special three-layer step-index fiber for building compact STED systems

**DOI:** 10.1038/s41598-019-44905-w

**Published:** 2019-06-11

**Authors:** Hao Luo, Guiren Wang, Libo Yuan

**Affiliations:** 1 Key Laboratory of In-Fiber Integrated Optics, Ministry of Education, College of Science, Harbin Engineering University, Harbin, 150001 People’s Republic of China; 2 Department of Mechanical Engineering and Biomedical Engineering Program, University of South Carolina, Columbia, SC 29208 USA; 3 Photonics Research Center, Guilin University of Electronics Technology, Guilin, 541004 People’s Republic of China

**Keywords:** Super-resolution microscopy, Nanoscale biophysics

## Abstract

Up to now, most of stimulated-emission-depletion (STED) systems were lens-based bulky systems. Exchanging some spatial light paths with optical fiber components will make the systems more flexible and will benefit various fields. A big problem to achieve this goal is that the STED beam generated by the traditional method of bulky systems cannot be maintained in an optical fiber due to its birefringence. In this article, we will introduce a type of special optical fiber. With the special fiber, a dark hollow beam with doughnut-shaped focal spot and a concentric beam with Gaussian-shaped focal spot can be generated at the same time. Parameters of a sample and a compact STED system based on it are demonstrated.

## Introduction

Stimulated-emission-depletion (STED) microscopy, since its discovery in the 1990s, has been widely studied and implemented in various of fields^1–10^. Thanks to its non-invasive method to break the diffraction-limited resolution barrier in far-field fluorescence microscopy, much more details can be obtained in biomedical studies *in vivo*, which promotes the progress of life-science^11–15^. In a typical STED microscopy system, an excitation beam that has a Gaussian-shaped focal spot would be overlapped with a STED beam that has a doughnut-shaped focal spot. The fluorophores settled in the STED doughnut area will be depleted and unable to do spontaneous emission. Because the dark center area of the STED doughnut can be infinitely small in theory, a fluorophore which is smaller than the diffraction limit of excitation beam can be distinguished in a STED microscopy system. The resolution of a STED microscope is commonly given by Eq. (1):

1 $$\Delta r\approx \frac{\lambda }{2NA\sqrt{1+{I}_{\max }/{I}_{s}}}$$

where *λ* is the wavelength of STED beam, *NA* is the numerical aperture of the objective lens, *I*_max_ is the maximum intensity of the doughnut-shaped STED focal spot, and *I*_*s*_ is the saturation intensity of the dye.

Up to now, most of STED microscopy systems are relatively complex and expensive. The doughnut-shaped focal spots are always generated by adding a phase vortex of appropriate handedness and topological charge on a circularly-polarized beam. This process requires appropriate wave plates, spiral phase plate, and precise optical paths^1–4,16^. Furthermore, to align the STED beam with the excitation beam concentrically is also a hard work in typical STED systems. It seems that if we can use some optical-fiber components to achieve the requirements of STED system, the system will become more compact and flexible.

Although the doughnut-shaped beam generated by the traditional method in bulky STED systems cannot be maintained in a length of optical fiber due to its birefringence, several methods have been reported to achieve doughnut-shaped beam directly from optical fibers^8,17–22^. However, these methods still require some specific modulations of incident light (e.g. modulations of phase and polarization state), which inevitably increases the complexity of system. In this article, we propose a special three-layer step-index fiber. A dark hollow beam with doughnut-shaped focal spot can be generated by this fiber. Besides, a concentric Gaussian-shaped light beam at another wavelength can be output at the same time. A compact STED fiber system was designed based on this special fiber.

## Results

### Characteristics of the special fiber

**Figure 1:**
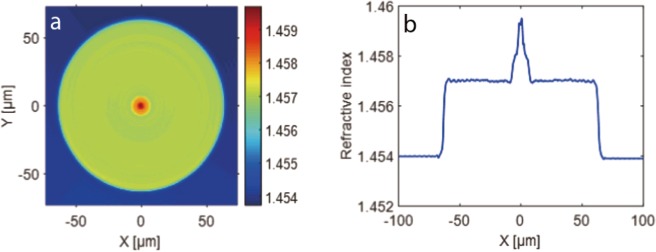
Refractive index of the special fiber. (**a**) Measured refractive index distribution on the cross-section of fiber. (**b**) The data along a diameter of (**a**).

This kind of special optical fiber has a three-layer structure. Its inner-cladding is very small, just similar as the size of core of normal communication optical fibers. And the diameter of the core of this special fiber is about one third of its inner-cladding. Traditional fiber mode system based on two-layer structure is no longer applicable in this kind of fibers^23,24^. A significant characteristic is that the inner-cladding of this special fiber can support guided modes as well as its core can. An approximate solution of mode system for this kind of fiber is deduced in the supplementary. In this article, we will introduce an example of this kind of special fiber, which can generate a dark hollow beam when a laser beam at the wavelength of 633 nm is input. The refractive index profile of this special fiber is shown in Fig. 1. The diameter of its core, inner-cladding and outer-cladding are about 4 μm, 13 μm, 125 μm, respectively. And the refractive indexes of them are about 1.4595, 1.458, 1.457, respectively.

**Figure 2:**
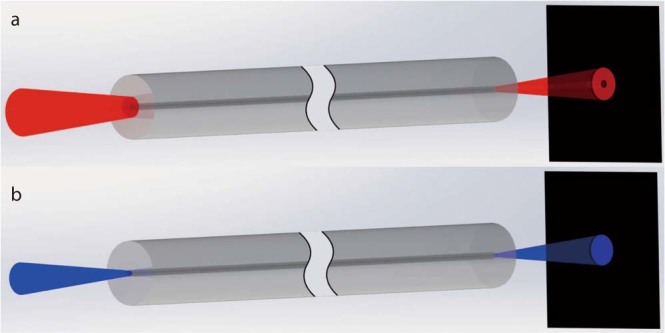
Output characteristic of the special fiber. (**a**) When a Gaussian-shaped laser beam at the wavelength of 633 nm is input into this fiber, whose focal spot can cover the inner-cladding of fiber, the output is a dark hollow beam. (**b**) When a laser beam at the wavelength of 475 nm is input into this fiber, whose focal spot can only cover the core of fiber, the output is a Gaussian beam.

When a laser beam at the wavelength of 633 nm is launched into this fiber, we can easily get a dark hollow beam as output. In experiments, we have tried three types of laser input: a normal Gaussian-shaped laser beam, focused by 40x objective; a dark-hollow beam generated by vortex phase plate, focused by 40x objective; and a beam directly output from a length of single-mode communication fiber. A decent dark hollow beam can be generated by the special fiber under each of the three incident conditions. The experimental results indicated that if only the incident light satisfied the condition that inner-cladding of the fiber can receive enough optical power (as shown in Fig. 2(a)) the output of special fiber would tend to have a dark center. A reasonable explanation of this characteristic in fiber-mode theory is as followed. While light is propagating in the fiber, guided modes with different propagation constants interfere. Under certain conditions, the optical power will be mainly coupled into a class of cylindrically-polarized modes at the output-end of fiber. Which leads to a dark center of the output light beam. More details of the mode analysis will be demonstrated in Methods section.

On the other hand, when the incident laser beam is focused small enough that main optical power of incident light is launched into the core of the fiber, the output beam will be typical Gaussian-shaped. As shown in Fig. 2(b). It seems that when the incident focal spot is small enough, the behavior of this special fiber degrades into a normal two-layer single-mode fiber. Most of the optical power is confined in the core while transmitting in the fiber. For every visible light, only the fundamental modes can be supported in core of the special fiber (more details will be shown in Methods section). Consequently, the output light beam is Gaussian-shaped. That is an interesting feature, since we can use this special fiber to generate a doughnut-shaped STED beam and a Gaussian-shaped excitation beam at the same time. Furthermore, these two beams will be concentric naturally as output.

Output characteristics of this special fiber depend heavily on the wavelength of incident light because fiber mode system will change as incident wavelength changes. In experiments, when the wavelength of incident light is chosen as 475 nm or 532 nm, we tried various of incident conditions, but could not get a doughnut-shaped distribution on cross-section of the output beam. However, a Gaussian-shaped output beam is easy to be obtained at any incident wavelength. As mentioned above.

**Figure 3:**
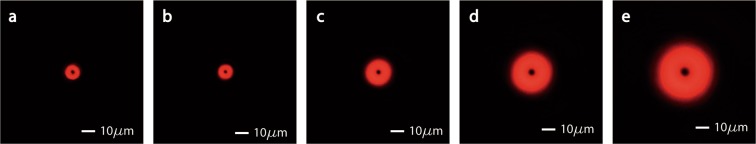
Optical power distributions on the cross-section of generated dark hollow beam at different distances from output-end of the special fiber. (**a**) Is the optical power distribution just at the fiber output-end. From (**b**) to (**e**), the distance between fiber output-end and the visual plane increased from 100 μm to 400 μm, with a step of 100 mm.

For a laser beam at the wavelength of 633 nm, when incident conditions are suitable that a doughnut-shaped beam is generated, the dark center in the cross-section of the beam can maintain a long distance, from near-field to far-field. Figure 3 shows the cross-sections of the output beam at different distances from output-end of the special fiber.

**Figure 4:**
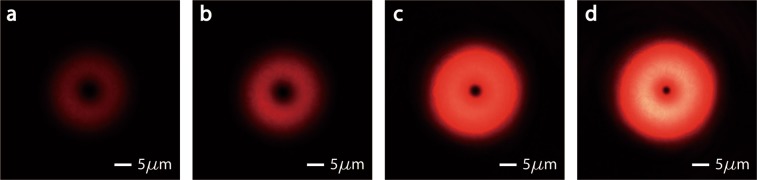
Optical power distribution on the cross-section varies with incident power. The photos are taken at a plane 260 μm away from the output-end of special fiber. From (**a**) to (**d**), we increased the input power gradually. As the power increasing, the ring became brighter and wider, and the dark center became smaller. The size of dark center can be infinitely small theoretically.

The size of dark center on the cross-section depends on the power of incident light. As shown in Fig. 4. When we increased the power of incident light, the distribution on the same cross-section varied: the red ring became brighter and wider, and the dark center became smaller. The size of dark center can be infinitely small if only the incident power is proper.

**Figure 5:**
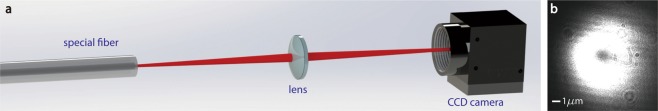
Optical power distribution on a cross-section (not focal plane) of the focused beam. (**a**) Shows a schematic diagram of the setup: the output light beam of special fiber was focused by a lens and then captured by a CCD camera. (**b**) Is a photo taken by CCD camera. It indicated the output light beam can maintain a decent doughnut-shaped cross-section when focused by lens.

In practice, the direct output of the special fiber is not suitable to be implemented as STED beam of a STED microscopy system since the output light from a fiber is divergent. To improve the efficiency of the system, specially-designed fiber-lens(es) must be used to focus the excitation and STED beams. In experiment, we have tested the property of focused beam of the special fiber output. Figure 5(a) shows the setup of test system. Figure 5(b) is a photo taken by the CCD camera. The photo shows that the output beam of the special fiber can maintain a decent doughnut-shaped cross-section when focused by lens. Note that the photo was not taken at the focal plane of the focused beam, limited by the resolution of the CCD camera we used. However, experimental results indicated that the size of dark center of the focused beam also depends on the power of incident light. In other words, the size of dark center on the focal plane can also be infinitely small if the incident power is proper.

### A design of compact STED system based on the special fiber

**Figure 6:**
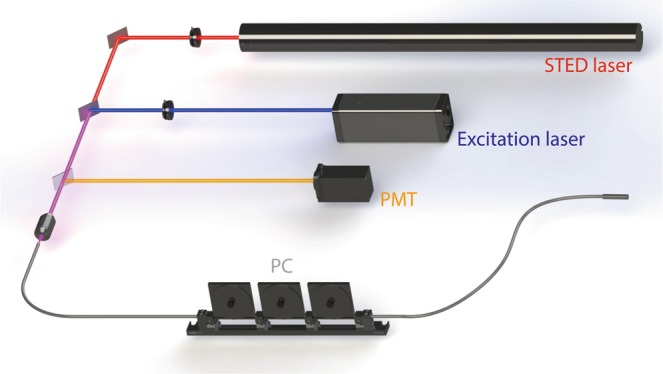
Experimental setup. Excitation laser is a CW laser at the wavelength of 475 nm. STED laser is a CW laser at the wavelength of 633 nm. PMT: Photomultiplier tube. PC: fiber polarization controller. Two diaphragms were used to adjust the size of incident focal spot. A fiber polarization controller was settled in the system to improve the quality of STED focal spot.

The design of a compact STED system is based on the special fiber we just introduced. We used a CW laser at the wavelength of 475 nm to generate the excitation beam, and another CW laser at the wavelength of 633 nm to generate the STED beam. As mentioned above, the size of incident focal spot will influence the output characteristic of this special fiber. Hence two diaphragms were set in front of the laser sources to adjust the size of incident laser beams. The whole system is very simple and concise because no pre-modulation of phase or polarization state is needed. In experiments, we found that the quality of the doughnut-shaped focal spot can be improved by stretching and twisting the fiber properly. Therefore, a polarization controller (PC) was implemented in the compact STED system to adjust the output pattern of the special fiber. The experimental setup is shown in Fig. 6.

**Figure 7:**
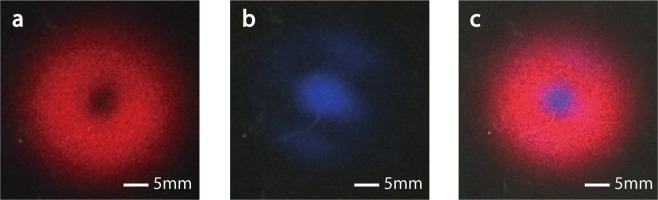
The far-field output patterns of the compact STED system. (**a**) Only 633 nm laser is input. (**b**) Only 475 nm laser is input. (**c**) Lasers at the two wavelengths are input together. In the three photos are projections of output light on a far-field screen.

Experimental results showed that the generated excitation beam and STED beam are aligned well naturally from the output-end of the special fiber. The two beams are concentric and parallel. Figure 7 shows three pictures of projections on a screen about a quarter of meter away from the output-end of the special fiber. In the far-field projections, the STED beam still has a decent doughnut-shaped pattern. Besides, the concentricity of STED beam and excitation beam keeps very high.

In a typical STED microscopy system, the selection of fluorophore and wavelengths of excitation and STED beams should satisfy the following principles: excitation wavelength should be near the peak wavelength of the excitation spectrum of the fluorophore to ensure better absorption; STED wavelength should be chosen at the tail of the long wave emission spectrum of the fluorophore to avoid the secondary excitation of the sample; the wavelength difference between excitation and STED beam should be wide enough to leave a comfortable spectral window for fluorescence detection. Several fluorophores that have been reported in STED microscopy systems are suitable for this pair of excitation beam (475 nm) and STED beam (633 nm) in theory. Such as Atto 532, DY-485XL, Chromeo 488, etc.^4,25–28^.

## Discussion

**Figure 8:**
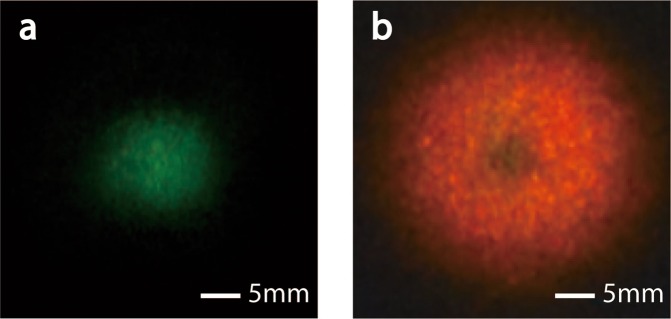
The output pattern of the special fiber projected on a screen: (**a**) only 532 nm laser is input; (**b**) lasers at the wavelengths of 532 nm and 633 nm are input together.

In the compact STED microscopy system we designed above, a laser source at the wavelength of 475 nm was used to generate the excitation beam. In fact, the wavelength of excitation beam is optional because the output light can be Gaussian-shaped for every visible light when the spot of incident light is small enough. For example, experimental results showed that a CW laser at the wavelength of 532 nm is also suitable to generate excitation beam for the STED system. Figure 8(a) showed the output projection of the special fiber on a screen, when a laser beam at the wavelength of 532 nm is input mainly in the core. And Fig. 8(b) is the projection when two laser beams at the wavelength of 633 nm and 532 nm are input into the fiber at the same time.

Several fluorophores suitable for this pair of excitation beam (475 nm) and STED beam (633 nm) have also been reported. Such as Atto 565, and NK51, etc.^26,29^.

In addition, the special fiber we demonstrated in this article is only an example of the special three-layer-structured fiber. Another special fiber which can generate doughnut-shaped beam at the wavelength of 532 nm has been obtained in our lab already. We expect that special fibers for any visible wavelength to generate doughnut-shaped beam can be developed through rational design in the future.

To increase the efficiency of the compact STED system in the future, special fiber lenses should be designed to reduce the insertion loss and optimize the power distribution of focal spot.

## Methods

### Mode analysis of the special fiber

**Figure 9:**
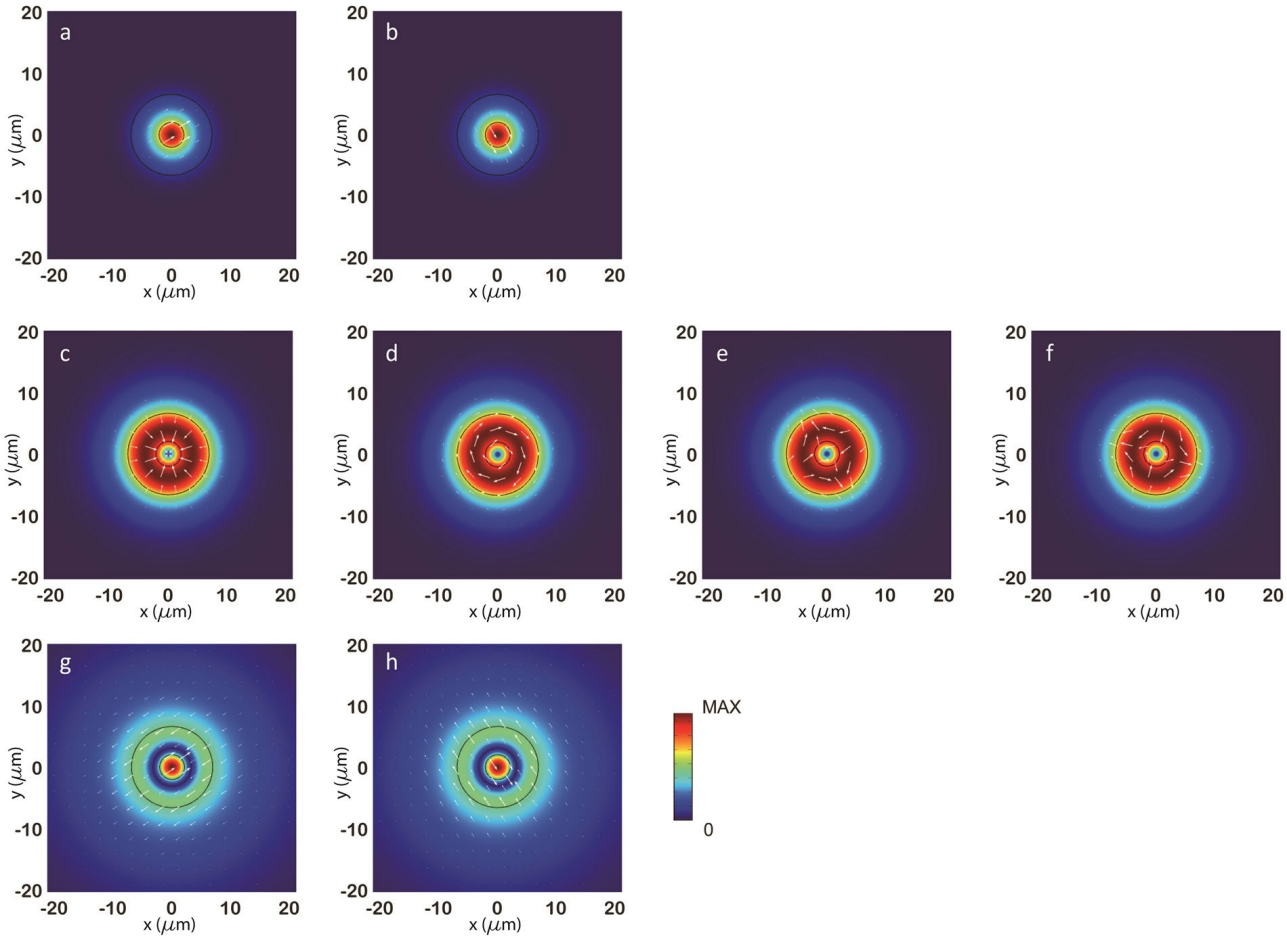
Mode system of the special fiber with incident wavelength of 633 nm. These simulation results are based on Finite Element Method. Three classes of guided modes in core and inner-cladding were obtained. The white arrows show the direction of electric field. (**a**,**b**) Are two degenerated modes with the effective refractive index of 1.4582. (**c**–**f**) Are four degenerated modes with the effective refractive index of 1.4574. (**g**,**h**) Are two degenerated modes with the effective refractive index of 1.4571.

To clearly analyze the mode-coupling phenomenon in the special fiber, numerical solutions of the mode system in this special fiber were obtained by Finite Element Method (commercial software: COMSOL Multiphysics). All the permitted guided modes in core and inner-cladding of the fiber at the wavelength of 633 nm are shown in Fig. 9. These guided modes can be divided into three classes by their effective refractive indices (or propagation constants). In Fig. 9(a,b) are in the first class with the effective refractive index of 1.4582; (c)(d)(e)(f) are in the second class with the effective refractive index of 1.4574; (g) and (h) are in the third class with the effective refractive index of 1.4571.

Simulation results showed that the field distributions of modes in the first class and modes in the third class are mainly constricted in core of the special fiber. Besides, modes in these two classes have similar polarization states. Under certain conditions, modes in the first class and modes in the third class will have a destructive interference in fiber core. At that time, main power of propagating light will be coupled to modes in the second class. Considering that modes in the second class are all cylindrically-polarized (see Fig. 9(c–f)), the output light beam will have a dark center consequently.

**Figure 10:**
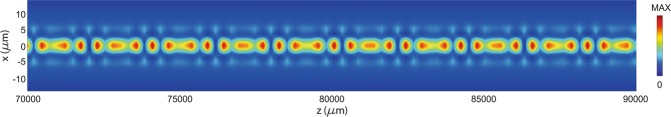
Simulation result showing the power-coupling phenomenon in the special fiber. The simulation result is based on Beam Propagation Method. Simulation is under such conditions: incident light beam is Gaussian beam at the wavelength of 633 nm; the beam is concentric with and perpendicular to the input-end of fiber; the diameter of the beam is 40 μm.

Another simulation based on Beam Propagation Method (commercial software: RSoft BPM) might show the power-coupling process in the special fiber more clearly. Figure 10 is the optical power distribution on the longitudinal-section of special fiber. The simulation result indicated that there is power-coupling between in core and in inner-cladding of the fiber alternatively while light is propagating in the fiber. At some certain distances, optical power will be mainly concentrated in inner-cladding and leave a dark center in core. As analyzed above, at these distances, main power of the transmitting light belongs to cylindrically-polarized modes. If the output-end of the special fiber is just at one of these distances, the output beam will be doughnut-shaped.

From Fig. 3(a), we have known that when the output beam is doughnut-shaped, main power is concentrated in inner-cladding of the special fiber at the output-end. Which is in accordance with the theory.

According to the above analyses, the ability of generating doughnut-shaped beam depends heavily on the length of special fiber. In experiments, we found the length of special fiber is indeed a factor to influence the property of output beam. However, experimental results also indicated that twisting and stretching of the fiber will stimulate the process of mode-coupling and influence the property of output beam. Hence, we have implemented a fiber polarization controller in the compact STED system as mentioned above. Fiber sections of different random lengths from about 1 m to 10 m were tested. By adjusting the fiber polarization controller settled in the fiber path (as shown in Fig. 6), we can always get an output beam with decent doughnut-shaped focal spot.

On the other hand, experimental results showed that a slight movement of the fiber tail will not change the output pattern dramatically. So that it is feasible to implement this special fiber in a scanning system.

### Optimizing the size of incident focal spot

As mentioned above, the size of incident focal spot on the input-end of fiber will influence the output pattern of the special fiber, since incident conditions will influence the ratio of guided modes excited in the fiber. For example, if the spot of incident light is very small that only fundamental modes in core can be excited (Fig. 9(a,b)), there will not be mode interference at all. Hence the output characteristic will be similar as a normal single-mode fiber.

**Figure 11:**
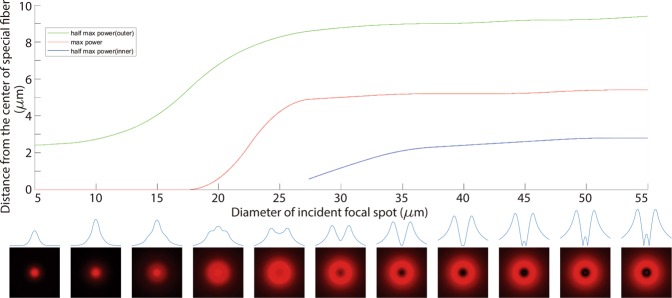
Variation tendency of optical power distribution on the output-end corresponding to the diameter of incident focal spot on the input-end of fiber: Green curve shows the distance between the center of fiber and the points with half max power (if the power distribution has a doughnut, choose the points on the outer ring). Red curve shows the distance between the center of fiber and the point(s) with max power. If the power distribution is doughnut-shaped, blue curve shows the distance between the center of fiber and the points with half max power on the inner ring. The pictures under the chart are simulated power distributions when diameter of incident focal spot varies from 5 μm to 55 μm, by a step of 5 μm.

Figure 11 shows the variation tendency of optical power distribution on the output-end of fiber. The simulation results are based on Beam Propagation Method (commercial software: RSoft BPM). Under an assumption that the incident beam is Gaussian-shaped, concentric with and perpendicular to the input-end of fiber. As mentioned above, the output pattern depends on the length of the special fiber. In these simulations, the length of the fiber is specially selected to satisfy that the optical power in core reaches a minimum value at the output end of fiber.

According to the simulation results, when the diameter of incident focal spot is less than 20 μm, the power distribution is not doughnut-shaped. When the diameter increases from 25 μm to 40 μm, the doughnut-shape of power distribution becomes increasingly obvious. When the diameter is more than 45 μm, a bright spot appears in the center of fiber, the power distribution is not doughnut-shaped again. To sum up, if a normal Gaussian-shaped laser beam is used as incident light, a decent doughnut-shaped power distribution can be achieved when the diameter is between 35 μm to 40 μm theoretically.

## Supplementary information

Mode analysis of the special fiber
