# Publisher Correction: A special three-layer step-index fiber for building compact STED systems

**DOI:** 10.1038/s41598-019-50176-2

**Published:** 2019-10-15

**Authors:** Hao Luo, Guiren Wang, Libo Yuan

**Affiliations:** 1 Key Laboratory of In-Fiber Integrated Optics, Ministry of Education, College of Science, Harbin Engineering University, Harbin, 150001 People’s Republic of China; 2 Department of Mechanical Engineering and Biomedical Engineering Program, University of South Carolina, Columbia, SC 29208 USA; 3 Photonics Research Center, Guilin University of Electronics Technology, Guilin, 541004 People’s Republic of China

Correction to: *Scientific Reports* 10.1038/s41598-019-44905-w, published online 11 June 2019

The original version of this Article contained a typographical error in the spelling of the author Hao Luo, which was incorrectly given as Hao Lou. This has now been corrected in the PDF and HTML versions of the Article.

