# Supplementary material for: A special three-layer step-index fiber for building compact STED systems
Source: Sci Rep. 2019 Jun 11;9:8455. doi: 10.1038/s41598-019-44905-w (PMC6560122; doi:10.1038/s41598-019-44905-w)
Supplement: Supplementary file 1 — Mode analysis of the special fiber [file 41598_2019_44905_MOESM1_ESM.docx]

**Supplementary: Mode analysis of the special fiber**

The refractive index of the three-layer step-index fiber is assumed as:

$n=\left\{\begin{matrix} n_{core},\text{ }0\leq r<r_{core} \\ n_{inner},\text{ }r_{core}\leq r<r_{inner} \\ n_{outer},\text{ }r\geq r_{inner} \end{matrix}\right.$ (1)

Where $n_{core}$, $n_{inner}$, $n_{outer}$ means the refractive index of core, inner-cladding, outer-cladding of fiber respectively. $r$ is the radial position, $r_{core}$ denotes the radius of core and $r_{inner}$ denotes the external radius of inner-cladding. In this model, we regard the external radius of outer cladding as infinite.

Analogous to traditional fiber mode theory, azimuthal and radial distribution of the electric and magnetic fields in the double-cladding fiber (DCF) should also meet the following equations respectively:

$\frac{d^{2}\Theta}{d\Theta^{2}}+m^{2}\Theta=0$ (2)

$\frac{d^{2}R}{dr^{2}}+\frac{1}{r}\cdot\frac{dR}{dr}+\left(\chi^{2}-\frac{m^{2}}{r^{2}}\right)R=0$ (3)

where $\Theta$ means the factor of electric or magnetic field components dependent on the azimuthal position$\theta$. Due to the periodicity of fiber cross-section, $m$ must be an integer. $R$ means the factor of electric or magnetic field components depended on the radial position $r$. Note that equation (3) is a Bessel equation and $m$ denotes the order of it. $\chi$ is a parameter which is described as:

$\chi^{2}\text{=}\left\{\begin{matrix} n_{core}^{2}k_{0}^{2}-\beta^{2},\text{ }0\leq r\leq r_{core} \\ n_{inner}^{2}k_{0}^{2}-\beta^{2},\text{ }r_{core}\leq r\leq r_{inner} \\ n_{outer}^{2}k_{0}^{2}-\beta^{2},\text{ }r\geq r_{inner} \end{matrix}\right.$ (4)

Here $k_{0}\text{=}\frac{2\pi}{\lambda}$ is the propagation constant of incident light in vacuum. $\lambda$ is the wavelength of incident light.

We can still regard the radial distribution of electromagnetic fields in core as a superposition of Bessel function of the first kind, and in outer-cladding as a superposition of modified Bessel function of the second kind, because of the physical reality that the amplitude of electromagnetic fields should not be infinite at the center of fiber and at infinity. In the inner-cladding, the electromagnetic field may have a similar behavior as that in the core. However, a significant difference is that we do not need to consider the infinite value of the Bessel function of the second kind at the center of fiber, since the center point is not included in the inner-cladding layer. In addition, because only the integral-order Bessel function of the first kind is insufficient to constitute a basic system of solutions of Eq. 3, we assume that the radial distribution of electromagnetic fields in the inner-cladding is a superposition of Bessel functions of the first kind and the second kind. Hence, we can get the shape of $E_{z}$ and $H_{z}$ as follow:

$E_{z}=\left\{\begin{matrix} A_{core}\cdot J_{m}\left(\frac{U_{core}\cdot r}{r_{core}}\right)\cdot e^{im\theta},\text{ }0\leq r\leq r_{core} \\ A_{inner1}\cdot J_{m}\left(\frac{U_{inner}\cdot r}{r_{inner}}\right)\cdot e^{im\theta}+A_{inner2}\cdot Y_{m}\left(\frac{U_{inner}\cdot r}{r_{inner}}\right)\cdot e^{im\theta},\text{ }r_{core}\leq r\leq r_{inner} \\ C\cdot K_{m}\left(\frac{W\cdot r}{r_{inner}}\right)\cdot e^{im\theta},\text{ }r\geq r_{inner} \end{matrix}\right.$ (5)

$H_{z}=\left\{\begin{matrix} B_{core}\cdot J_{m}\left(\frac{U_{core}\cdot r}{r_{core}}\right)\cdot e^{im\theta},\text{ }0\leq r\leq r_{core} \\ B_{inner1}\cdot J_{m}\left(\frac{U_{inner}\cdot r}{r_{inner}}\right)\cdot e^{im\theta}+B_{inner2}\cdot Y_{m}\left(\frac{U_{inner}\cdot r}{r_{inner}}\right)\cdot e^{im\theta},\text{ }r_{core}\leq r\leq r_{inner} \\ D\cdot K_{m}\left(\frac{W\cdot r}{r_{inner}}\right)\cdot e^{im\theta},\text{ }r\geq r_{inner} \end{matrix}\right.$ (6)

Where $A_{core}$, $A_{inner1}$, $A_{inner2}$, $C$, $B_{core}$, $B_{inner1}$, $B_{inner2}$, $D$ are eight undetermined coefficients which can be determined from boundary conditions. $U_{core}$, $U_{inner}$, $W$are determined by:

$U_{core}^{2}=\left(k_{0}^{2}n_{core}^{2}-\beta^{2}\right)r_{core}^{2}$ (7)

$U_{inner}^{2}=\left(k_{0}^{2}n_{inner}^{2}-\beta^{2}\right)r_{inner}^{2}$ (8)

$W^{2}=\left(\beta^{2}-k_{0}^{2}n_{outer}^{2}\right)r_{inner}^{2}$ (9)

According to the relationship among the components of electromagnetic fields in fiber in the cylindrical coordinates system:

$E_{r}=-\frac{i}{\chi^{2}}\left(\beta\cdot\frac{\partial E_{z}}{\partial r}+\frac{\omega\mu_{0}}{r}\cdot\frac{\partial H_{z}}{\partial\theta}\right)$ (10)

$E_{\theta}=-\frac{i}{\chi^{2}}\left(\frac{\beta}{r}\cdot\frac{\partial E_{z}}{\partial\theta}-\omega\mu_{0}\cdot\frac{\partial H_{z}}{\partial r}\right)$ (11)

$H_{r}=-\frac{i}{\chi^{2}}\left(\beta\cdot\frac{\partial H_{z}}{\partial r}-\frac{\omega\varepsilon}{r}\cdot\frac{\partial E_{z}}{\partial\theta}\right)$ (12)

$H_{\theta}=-\frac{i}{\chi^{2}}\left(\frac{\beta}{r}\cdot\frac{\partial H_{z}}{\partial\theta}+\omega\varepsilon\cdot\frac{\partial E_{z}}{\partial r}\right)$ (13)

where $\omega$ is the frequency of incident light, $\mu_{0}$ is the permeability in vacuum, and $\varepsilon$ is the electrical conductivity of fiber materials, defined as:

$\varepsilon=\left\{\begin{matrix} \varepsilon_{core},\text{ 0}\leq\text{r < r}_{core} \\ \varepsilon_{inner},\text{ r}_{core}\leq r<r_{inner} \\ \varepsilon_{outer},\text{ r}\geq\text{r}_{inner} \end{matrix}\right.$ (14)

where $\varepsilon_{core}$, $\varepsilon_{inner}$, $\varepsilon_{outer}$ are the electrical conductivity of the core, inner-cladding and outer-cladding, respectively. Using Eq. (5), (6), (11), (13), we can get that:

$\begin{matrix} E_{\theta}= \\ \left\{\begin{matrix} -i\left(\frac{r_{core}}{U_{core}}\right)^{2}\left[\frac{im\beta}{r}\cdot A_{core}\cdot J_{m}\left(\frac{U_{core}\cdot r}{r_{core}}\right)-\omega\mu_{0}\cdot\left(\frac{U_{core}}{r_{core}}\right)\cdot B_{core}\cdot J_{m}'\left(\frac{U_{core}\cdot r}{r_{core}}\right)\right]\cdot e^{im\theta},\text{ }0\leq r\leq r_{core}\text{ } \\ -i\left(\frac{r_{inner}}{U_{inner}}\right)^{2}\left\{\begin{matrix} \frac{im\beta}{r}\cdot\left[A_{inner1}\cdot J_{m}\left(\frac{U_{inner}\cdot r}{r_{inner}}\right)+A_{inner2}\cdot Y_{m}\left(\frac{U_{inner}\cdot r}{r_{inner}}\right)\right] \\ -\omega\mu_{0}\cdot\left(\frac{U_{inner}}{r_{inner}}\right)\cdot\left[B_{inner1}\cdot J_{m}'\left(\frac{U_{inner}\cdot r}{r_{inner}}\right)+B_{inner2}\cdot Y_{m}'\left(\frac{U_{inner}\cdot r}{r_{inner}}\right)\right] \end{matrix}\right\}\cdot e^{im\theta},r_{core}\leq r\leq r_{inner} \\ i\left(\frac{r_{inner}}{W}\right)^{2}\left[\frac{im\beta}{r}\cdot C\cdot K_{m}\left(\frac{W\cdot r}{r_{inner}}\right)-\omega\mu_{0}\cdot\left(\frac{W}{r_{inner}}\right)\cdot D\cdot K_{m}'\left(\frac{W\cdot r}{r_{inner}}\right)\right]\cdot e^{im\theta},\text{ }r\geq r_{inner} \end{matrix}\right. \end{matrix}$(15)

$\begin{matrix} H_{\theta}= \\ \left\{\begin{matrix} -i\left(\frac{r_{core}}{U_{core}}\right)^{2}\left[\frac{im\beta}{r}\cdot B_{core}\cdot J_{m}\left(\frac{U_{core}\cdot r}{r_{core}}\right)+\omega\varepsilon_{core}\cdot\left(\frac{U_{core}}{r_{core}}\right)\cdot A_{core}\cdot J_{m}'\left(\frac{U_{core}\cdot r}{r_{core}}\right)\right]\cdot e^{im\theta},\text{ }0\leq r\leq r_{core}\text{ } \\ -i\left(\frac{r_{inner}}{U_{inner}}\right)^{2}\left\{\begin{matrix} \frac{im\beta}{r}\cdot\left[B_{inner1}\cdot J_{m}\left(\frac{U_{inner}\cdot r}{r_{inner}}\right)+B_{inner2}\cdot Y_{m}\left(\frac{U_{inner}\cdot r}{r_{inner}}\right)\right] \\ +\omega\varepsilon_{inner}\cdot\left(\frac{U_{inner}}{r_{inner}}\right)\cdot\left[A_{inner1}\cdot J_{m}'\left(\frac{U_{inner}\cdot r}{r_{inner}}\right)+A_{inner2}\cdot Y_{m}'\left(\frac{U_{inner}\cdot r}{r_{inner}}\right)\right] \end{matrix}\right\}\cdot e^{im\theta},r_{core}\leq r\leq r_{inner} \\ i\left(\frac{r_{inner}}{W}\right)^{2}\left[\frac{im\beta}{r}\cdot D\cdot K_{m}\left(\frac{W\cdot r}{r_{inner}}\right)+\omega\varepsilon_{outer}\cdot\left(\frac{W}{r_{inner}}\right)\cdot C\cdot K_{m}'\left(\frac{W\cdot r}{r_{inner}}\right)\right]\cdot e^{im\theta},\text{ }r\geq r_{inner} \end{matrix}\right. \end{matrix}$ (16)

Considering the continuity of $E_{z}$, $H_{z}$, $E_{\theta}$, and $H_{\theta}$ on the boundaries at $r=r_{core}$ and $r=r_{inner}$, we can get eight equations for the eight undetermined coefficients. So that these coefficients can be determined theoretically.

However, the eight equations are very complicated and are transcendental. Therefore, we propose to take some approximations first. Based on the weakly-guiding approximation, we can treat the refractive index of the three layers as the same. That is:

$n_{core}=n_{inner}=n_{outer}$ (17)

According to Eq. (17), (7), (8) and that $n=\sqrt{\varepsilon\mu}$, we take a further step and assume that：

$U_{core}=U_{inner}$ (18)

$\varepsilon_{core}=\varepsilon_{inner}=\varepsilon_{outer}$ (19)

Under these approximations, using the boundary conditions of $E_{z}$, $H_{z}$, we can get that:

$\left\{\begin{matrix} A_{core}=\frac{A_{inner1}\cdot J_{m}\left(\frac{U_{inner}\cdot r_{core}}{r_{inner}}\right)+A_{inner2}\cdot Y_{m}\left(\frac{U_{inner}\cdot r_{core}}{r_{inner}}\right)}{J_{m}\left(U_{core}\right)} \\ C=\frac{A_{inner1}\cdot J_{m}\left(U_{inner}\right)+A_{inner2}\cdot Y_{m}\left(U_{inner}\right)}{K_{m}\left(W\right)} \\ B_{core}=\frac{B_{inner1}\cdot J_{m}\left(\frac{U_{inner}\cdot r_{core}}{r_{inner}}\right)+B_{inner2}\cdot Y_{m}\left(\frac{U_{inner}\cdot r_{core}}{r_{inner}}\right)}{J_{m}\left(U_{core}\right)} \\ D=\frac{B_{inner1}\cdot J_{m}\left(U_{inner}\right)+B_{inner2}\cdot Y_{m}\left(U_{inner}\right)}{K_{m}\left(W\right)} \end{matrix}\right.$ (20)

Substitute Eq. (20) into Eq. (15) and (16), with our approximations, we can get that:

$\left(\begin{matrix} M_{11} & M_{12} & M_{13} & M_{14} \\ M_{21} & M_{22} & M_{23} & M_{24} \\ 0 & M_{32} & 0 & 0 \\ 0 & 0 & 0 & M_{44} \end{matrix}\right)\left(\begin{matrix} A_{inner1} \\ A_{inner2} \\ B_{inner1} \\ B_{inner2} \end{matrix}\right)=0$ (21)

The elements in that matrix are defined as:

$$M_{11}=im\beta\cdot\left(\frac{1}{U_{inner}^{2}}+\frac{1}{W^{2}}\right)\cdot J_{m}\left(U_{inner}\right)$$

$$M_{12}=im\beta\cdot\left(\frac{1}{U_{inner}^{2}}+\frac{1}{W^{2}}\right)\cdot Y_{m}\left(U_{inner}\right)$$

$$M_{13}=-\omega\mu_{0}\left[\frac{J_{m}'\left(U_{inner}\right)}{U_{inner}^{2}}+\frac{K_{m}'\left(W\right)\cdot J_{m}\left(U_{inner}\right)}{W^{2}\cdot K_{m}\left(W\right)}\right]$$

$$M_{14}=-\omega\mu_{0}\left[\frac{Y_{m}'\left(U_{inner}\right)}{U_{inner}^{2}}+\frac{K_{m}'\left(W\right)\cdot Y_{m}\left(U_{inner}\right)}{W^{2}\cdot K_{m}\left(W\right)}\right]$$

$$M_{21}=\omega\varepsilon_{inner}\left[\frac{J_{m}'\left(U_{inner}\right)}{U_{inner}^{2}}+\frac{K_{m}'\left(W\right)\cdot J_{m}\left(U_{inner}\right)}{W\cdot K_{m}\left(W\right)}\right]$$

$$M_{22}=\omega\varepsilon_{inner}\left[\frac{Y_{m}'\left(U_{inner}\right)}{U_{inner}^{2}}+\frac{K_{m}'\left(W\right)\cdot Y_{m}\left(U_{inner}\right)}{W\cdot K_{m}\left(W\right)}\right]$$

$$M_{23}=im\beta\cdot\left(\frac{1}{U_{inner}^{2}}+\frac{1}{W^{2}}\right)\cdot J_{m}\left(U_{inner}\right)$$

$$M_{24}=im\beta\cdot\left(\frac{1}{U_{inner}^{2}}+\frac{1}{W^{2}}\right)\cdot Y_{m}\left(U_{inner}\right)$$

$$M_{32}=\varepsilon_{inner}\cdot\left[Y_{m}'\left(\frac{U_{inner}\cdot r_{core}}{r_{inner}}\right)-\frac{J_{m}'\left(\frac{U_{inner}\cdot r_{core}}{r_{inner}}\right)\cdot Y_{m}\left(\frac{U_{inner}\cdot r_{core}}{r_{inner}}\right)}{J_{m}\left(\frac{U_{inner}\cdot r_{core}}{r_{inner}}\right)}\right]$$

$$M_{44}=Y_{m}'\left(\frac{U_{inner}\cdot r_{core}}{r_{inner}}\right)-\frac{J_{m}'\left(\frac{U_{inner}\cdot r_{core}}{r_{inner}}\right)\cdot Y_{m}\left(\frac{U_{inner}\cdot r_{core}}{r_{inner}}\right)}{J_{m}\left(\frac{U_{inner}\cdot r_{core}}{r_{inner}}\right)}$$

For guided mode in the fiber, $A_{inner1}$, $A_{inner2}$, $B_{inner1}$, and $B_{inner2}$ should not be completely zero. Hence, the determinant of the matrix in Eq. (21) must be zero. After simplification, we can get that:

$\left[\frac{J_{m}'\left(U_{inner}\right)}{U_{inner}^{2}}+\frac{K_{m}'\left(W\right)\cdot J_{m}\left(U_{inner}\right)}{W^{2}\cdot K_{m}\left(W\right)}\right]\cdot\left[Y_{m}'\left(\frac{U_{inner}\cdot r_{core}}{r_{inner}}\right)-\frac{J_{m}'\left(\frac{U_{inner}\cdot r_{core}}{r_{inner}}\right)\cdot Y_{m}\left(\frac{U_{inner}\cdot r_{core}}{r_{inner}}\right)}{J_{m}\left(\frac{U_{inner}\cdot r_{core}}{r_{inner}}\right)}\right]^{3}=0$ (22)

Eq. (22) can be split into two equations by factorization:

$\frac{J_{m}'\left(U_{inner}\right)}{U_{inner}^{2}}+\frac{K_{m}'\left(W\right)\cdot J_{m}\left(U_{inner}\right)}{W^{2}\cdot K_{m}\left(W\right)}$=0 (23a)

or

$Y_{m}'\left(\frac{U_{inner}\cdot r_{core}}{r_{inner}}\right)-\frac{J_{m}'\left(\frac{U_{inner}\cdot r_{core}}{r_{inner}}\right)\cdot Y_{m}\left(\frac{U_{inner}\cdot r_{core}}{r_{inner}}\right)}{J_{m}\left(\frac{U_{inner}\cdot r_{core}}{r_{inner}}\right)}=0$ (23b)

Note that in the third and fourth row of the coefficient matrix in Eq. (21) there is only one non-zero element, $M_{32}$and $M_{44}$ respectively. And $M_{32}$ is coincidentally $\varepsilon_{inner}$ times $M_{44}$. If Eq. (23b) is satisfied, $A_{inner2}$ and $B_{inner2}$ can be arbitrary values.

In this article, we will temporarily call the mode when Eq. (23a) or (23b) is satisfied as MC mode or MS mode, respectively.

Here, in analogy with the traditional fiber mode theory we define $V$ as:

$V^{2}=U_{inner}^{2}+W^{2}$ (24a)

With Eq. (8) and (9), we can get that:

$V^{2}=\left(n_{inner}^{2}-n_{outer}^{2}\right)\cdot k_{0}^{2}\cdot r_{inner}^{2}$ (24b)

$V$ is determined only by the parameters of the fiber and the incident light, which will guide the design of the fiber.

Considering the case when Eq. (23a) is satisfied. If $W\to0$, $U\to V$ , $\frac{K_{m}'\left(W\right)}{W^{2}\cdot K_{m}\left(W\right)}\to\infty$, and $\frac{J_{m}'\left(U_{inner}\right)}{U_{inner}^{2}}$approaches a finite value. If Eq. (23a) is satisfied, then $J_{m}\left(U_{inner}\right)\to0$. Therefore, the corresponding cut-off condition is:

$J_{m}\left(U_{cmn}\right)=0$ (25)

where $U_{cmn}$ means the eigenvalue when this kind of mode is cut off and *n* means the sequence number of the zero point of Bessel function.

Note that when *m*=1, the cut-off condition of MC_1n_ is $J_{1}\left(U_{c1n}\right)=0$. In this case, U_c11_=0, U_c12_=3.8317, U_c13_=7.0156. U_c11_=0 means that MC_11_ mode cannot be cut off.

Considering equation (23b), which depends only on $\frac{U_{inner}\cdot r_{core}}{r_{inner}}$. The cut-off condition is:

$Y_{m}'\left(U_{smn}\right)-\frac{J_{m}'\left(U_{smn}\right)\cdot Y_{m}\left(U_{smn}\right)}{J_{m}\left(U_{smn}\right)}=0$ (26)

$U_{smn}$ means the eigenvalue if $\frac{U_{inner}\cdot r_{core}}{r_{inner}}<U_{smn}$this kind of mode is cut off. *n* means the sequence number of the zero point of Bessel function.

Some cut-off conditions of low-order MC modes and MS modes are deduced by numerical calculations, as shown in table 1.

Table 1. Cut-off conditions of low-order MC and MS modes

|  | Cut-off condition |  | Cut-off condition |
| --- | --- | --- | --- |
| Mode name | $U_{inner}<$ | Mode name | $\frac{U_{inner}r_{core}}{r_{inner}}<$ |
| MC_11_ | 0 | MS_01_ | 0.8936 |
| MC_01_ | 2.4048 | MS_11_ | 2.1971 |
| MC_12_ | 3.8317 | MS_02_ | 2.4048 |
| MC_21_ | 5.1356 | MS_21_ | 3.3842 |
| MC_02_ | 5.5201 | MS_12_ | 3.8317 |
| MC_31_ | 6.3802 | MS_03_ | 3.9577 |
| MC_13_ | 7.0156 | MS_31_ | 4.5270 |
| MC_41_ | 7.5883 | MS_22_ | 5.1356 |
| MC_22_ | 8.4172 | MS_13_ | 5.4297 |
| MC_03_ | 8.6537 | MS_32_ | 6.3802 |
